# Leptospirosis in the Platypus (*Ornithorhynchus anatinus*) in Australia: Who Is Infecting Whom?

**DOI:** 10.3390/ani14192834

**Published:** 2024-10-01

**Authors:** Richard J. Whittington, Thomas R. Grant, Jarrad McKercher, Monica Suann, Keith Hart, Kathrine A. Handasyde, James Macgregor, Mark E. Westman, Joanne H. Connolly

**Affiliations:** 1 School of Veterinary Science, University of Sydney, 425 Werombi Road, Camden, NSW 2570, Australia; mark.westman@dpi.nsw.gov.au; 2 School of Biological, Earth and Environmental Sciences, University of New South Wales, Sydney, NSW 2052, Australia; t.grant@unsw.edu.au; 3 Centre for People, Place and Planet, Edith Cowan University, 270 Joondalup Drive, Joondalup, WA 6027, Australia; j.mckercher@ecu.edu.au; 4 Elizabeth Macarthur Agricultural Institute, Woodbridge Road, Menangle, NSW 2568, Australia; monica.suann@dpi.nsw.gov.au; 5 Pastures Protection Board Braidwood, Braidwood, NSW 2622, Australia; 6 BioSciences, The University of Melbourne, Parkville, VIC 3052, Australia; 7 UVC Wild, Ulverstone Veterinary Clinic, Ulverstone, TAS 7315, Australia; 8 Conservation Medicine Program, School of Veterinary Medicine, Murdoch University, Murdoch, WA 6150, Australia; 9 School of Agricultural, Environmental and Veterinary Sciences, Charles Sturt University, Locked Bag 588, Wagga Wagga, NSW 2678, Australia; jconnolly@csu.edu.au

**Keywords:** leptospirosis, *Leptospira interrogans* serovar Hardjo, platypus, *Ornithorhynchus anatinus*, epidemiology, spatial analysis

## Abstract

**Simple Summary:**

The platypus (*Ornithorhynchus anatinus*) is a monotreme, a rare kind of mammal that lays eggs and suckles its young. It is amphibious, lives in freshwater, and is found only in Australia, where it is closely protected. During prior conservation studies, several pathogens had been discovered in the platypus, including leptospires, which are a kind of bacterium that can cause severe disease in animals and people. We evaluated the available data concerning this infection in platypuses, tested blood samples in the Platypus Serum Bank, and combined the results with historical records. We found evidence of leptospirosis in 50% of platypuses from 14 river basins in southeastern Australia. Leptospiral infection did not stop females from breeding and juvenile platypuses were recruited to the population each year. The high seroprevalence, evidence of ongoing exposure to leptospires, lack of disease, mild kidney pathology, and visible leptospires in the kidney tubules suggest that the platypus may be a reservoir host for leptospires rather than an accidental host that suffers from the disease. Cattle on adjacent farms also had leptospirosis, but the evidence was not convincing that cattle caused the infection in platypuses. A landscape-wide study is recommended to determine the actual infection pathway.

**Abstract:**

The platypus (*Ornithorhynchus anatinus*) is an amphibious, egg-laying mammal of high conservation value that is found only in Australia. The zoonotic bacterium *Leptospira interrogans* serovar Hardjo was discovered in platypuses in prior studies, but little is known about its epidemiology. Samples in the Platypus Serum Bank were tested in 2023 and the results were combined with historical records. Antibodies against *L. interrogans* serovar Hardjo were found in 50% of 464 serum samples from 411 platypuses collected from 14 river basins in southeastern Australia between 1981 and 2012; prevalence remained high over three decades in the Shoalhaven River population. Seroprevalence increased with age, suggesting environmental exposure. Individual platypuses had persistent titres, some for six years. Seropositive females lactated, juveniles were recruited into the population, and there were no reports of clinical leptospirosis. Three necropsied platypuses were seropositive and had mild nephritis with leptospires in the renal tubules. The high seroprevalence, persistent titres, lack of disease, mild renal lesions, and renal colonisation suggest the platypus may be a maintenance host. Sympatric cattle had *L. interrogans* serovar Hardjo titres, but the spatial association with seropositive platypuses was statistically weak. Other mammalian wildlife species and sheep also have *L. interrogans* serovar Hardjo titres; therefore, a complex ecological network must be considered. A landscape-wide study is recommended to properly assess transmission pathways and confirm who is infecting whom.

## 1. Introduction

The platypus (*Ornithorhynchus anatinus*) is a semi-fossorial, amphibious, egg-laying mammal found only in Australia. It is a remnant of a more diverse fossil lineage from the southern continent of Gondwana and is the only surviving species in the family Ornithorhynchidae in the order Monotremata [1]. It occurs on the eastern part of the mainland of Australia and in Tasmania (TAS), including King Island, with an introduced population on Kangaroo Island [1]. Predominantly nocturnal, but also active around dawn and dusk, the platypus inhabits freshwater streams, rivers, shallow lakes, and wetlands, with earth banks being suitable for nesting and resting burrows, ideally consolidated by riparian vegetation [1]. These features can occur in natural bushland, agricultural, and peri-urban areas. It feeds mainly on benthic macroinvertebrates [1].

While not considered threatened nationally [2], the platypus is listed by the International Union for Conservation of Nature (IUCN) as Near Threatened [3]. It is listed as Vulnerable in Victoria (VIC) [4], and while it has never been common or widespread in South Australia, there it is considered Endangered [5,6]. Although reductions in some local populations have been reported in all states except for TAS [3], the species is still widely distributed over its historical range [5].

Assessments of diseases affecting platypuses have been conducted as part of long-term conservation biology studies [7,8,9]. Other initiatives include the Australian Registry of Wildlife Health that catalogues information (https://taronga.org.au/conservation-and-science/australian-registry-of-wildlife-health#:~:text=The%20Australian%20Registry%20of%20Wildlife,both%20passive%20and%20active%20surveillance) (accessed on 31 July 2024) and the Platypus Serum Bank at the University of Sydney that was established by the corresponding author with objectives to enable epidemiological studies [10].

Relatively few significant infectious diseases have been reported among platypuses, but leptospiral infection [11] stands out for assessment because it causes significant disease in other mammals and is an important zoonosis [12]. Leptospires are slender, helical, motile, Gram-negative bacteria with specific adaptations to survive in water and soil [13,14,15]. Pathogenic *Leptospira* species localise in the renal tubules of maintenance/reservoir hosts that shed them in urine, leading to the infection of accidental/incidental/spillover hosts that succumb to leptospirosis [16]. Cases may present with a subacute or an acute syndrome that can be fatal [17]. The pathogenesis of leptospirosis involves bacterial attachment to damaged skin or mucous membranes, rapid penetration and entry into the blood stream, a short bacteraemic phase during which symptoms may develop, and then localisation in a range of organs, resulting in clinical signs of the disease [16,18]. Serum antibodies develop after the commencement of leptospiral shedding in the urine, and the antibody response lasts months to years, accompanied by persistent leptospiruria [19]. Infection in maintenance hosts can be inapparent, but there is morbidity in some species, such as reproductive failure in cattle infected with *Leptospira interrogans* serovar Hardjo [20]. Leptospiral maintenance hosts exist across mammalian families, at a reported prevalence generally of about 15% and with some specificity of each host species for particular leptospiral species [21,22,23]. For example, *L. interrogans* serovar Icterohaemorrhagiae is maintained in the brown rat, *Rattus norvegicus*, while *L. interrogans* serovar Canicola is maintained in the pig, *Sus scrofa* [24].

Leptospiral infection was discovered in platypuses in the upper Shoalhaven River, New South Wales (NSW) in 1985 [11]. Almost half (47%) of the 17 platypuses sampled there were seropositive for *L. interrogans* serovar Hardjo. Antibody titres against *L. interrogans* serovar Hardjo were subsequently reported in platypuses from the Wollondilly River, NSW [25] and the Inglis River catchment in TAS [9]. *L. interrogans* serovar Hardjo commonly infects cattle in Australia. Consequently, McColl and Whittington [11] wondered whether there was a connection between *L. interrogans* serovar Hardjo infections in platypuses and those in cattle, especially because the two species can come into close contact along rivers. However, little is known about the epidemiology of leptospirosis in the platypus and the question of interspecies transfer has not yet been answered.

The platypus is a cryptic and closely protected species, and opportunities for research are very limited. The aims of this study, within the limits imposed by retrospective analysis of opportunistically collected samples, were (1) to determine the geographic distribution, prevalence, and persistence of leptospiral infections in platypus populations in southeastern Australia; (2) to describe the seropositivity to *Leptospira* according to the age and sex of the animals; and (3) to evaluate evidence of transmission of leptospiral infections between cattle and platypus. The study was enabled by the testing of samples in the Platypus Serum Bank, the aggregation of serological results from published reports, the collation and analysis of archival laboratory records for both platypuses and sympatric cattle, and contemporary spatial analysis. A surprising outcome was that the platypus may be a maintenance host for leptospires in *L. interrogans* serovar Hardjo.

## 2. Materials and Methods

### 2.1. General

Methods for animal sampling and all laboratory tests are provided, as they have not been published for this sample set. Results aggregated from two published reports [11,25], where included in specific analyses, are clearly identified.

### 2.2. Study Sites

**Figure 1:**
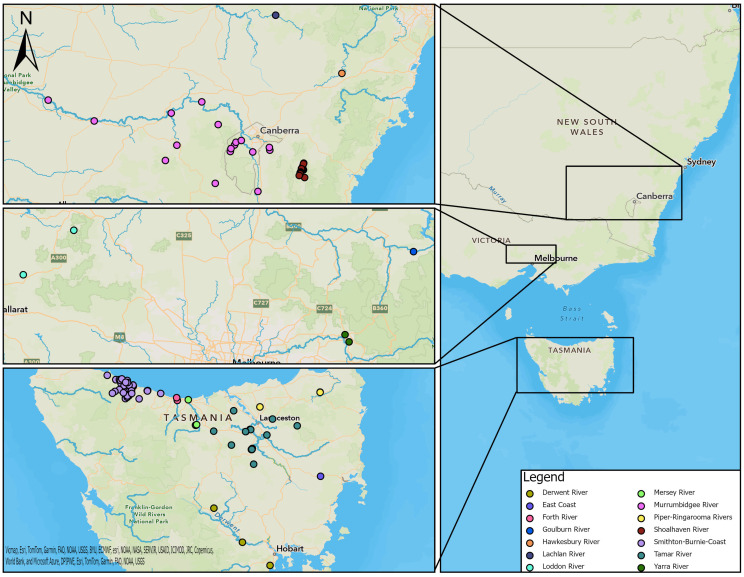
Locations of study sites in each river basin in southeastern Australia (**right**), including NSW and ACT (**top left**), VIC (**middle left**), and TAS (**bottom left**).

Samples were collected from platypuses in river basins and impoundments within 14 drainage divisions defined by the Australian Government [26] (Figure 1). Samples from captive platypuses in zoos were not assigned to a drainage division or river basin.

### 2.3. Trapping and Sampling from Free-Living Platypuses

Trapping and sampling were conducted by the authors unless otherwise stated in the Acknowledgments. Platypuses in NSW and the Australian Capital Territory (ACT) were caught in unweighted mesh nets [27], while those in TAS and VIC were also caught in fyke nets [28,29,30]. Animals were individually identified with numbered leg bands and/or microchips [27,31]. Sex and age were determined based on spur morphology [32,33]. In the first years of the study, animals in NSW as well as those captured in TAS in 2012 were anaesthetised prior to blood sampling, whereas others were conscious and restrained in a pillow case with the bill protruding from a hole cut in one corner [34,35]. The bill was swabbed with 70% ethanol. Blood was collected from the marginal sinus as previously described and illustrated [35,36]. Adult female platypuses captured in the upper Shoalhaven River between September and April were injected intramuscularly in the upper hind leg with 0.1–0.2 mL of oxytocin (1–2 International Units; Syntocinon, Novartis). After 5–10 min, the mammary area on the lower ventral abdomen was palpated. Milk ejected onto the fur was a proxy for the detection of females with dependent young in nesting burrows [37,38].

### 2.4. Routine Processing of Platypus Blood Samples

Blood was placed in plain glass tubes and allowed to clot at room temperature for 2 h or chilled until receipt at a laboratory. Clotted blood was centrifuged at 2000× *g* and the serum was harvested, transferred to tubes, and frozen (−20 °C).

### 2.5. Platypus Serum Bank

Depending on sampling location, samples were prepared for archiving either before or after the initial freeze. Serum for archiving was diluted at 1:10 in the cryoprotectant buffer TSGM (25 mM Tris-HCl pH 7.4, 0.15M NaCl, 50% *v*/*v* glycerol, 0.02% *w*/*v* merthiolate) in sterile 1.5 mL polypropylene screw capped tubes (Edwards Scientific, Narellan, NSW, Australia), allocated a unique number and placed at −20 °C; samples remained in a liquid state in this buffer. The archive freezer was monitored daily; during the storage period, there was one electrical failure with a duration of three days, when the storage temperature briefly reached 20 °C.

### 2.6. Necropsy Samples

Government authorities made carcases available for necropsies. These were performed at the Regional Veterinary Laboratory (RVL), Wagga Wagga, NSW. Blood was collected by means of cardiac puncture and processed as described above. The kidneys and urine aspirated from the bladder were collected for leptospiral cultures. Routine histopathological examination of the kidney was undertaken; tissues were fixed in 10% *v*/*v* buffered formalin, dehydrated through graded ethanol solutions, embedded in paraffin, and sectioned at 5 µm. Sections of the kidney were stained with haematoxylin and eosin (H&E) and Warthin–Starry silver [39] and examined via light microscopy.

### 2.7. Collection and Processing of Blood Samples from Cattle

**Figure 2:**
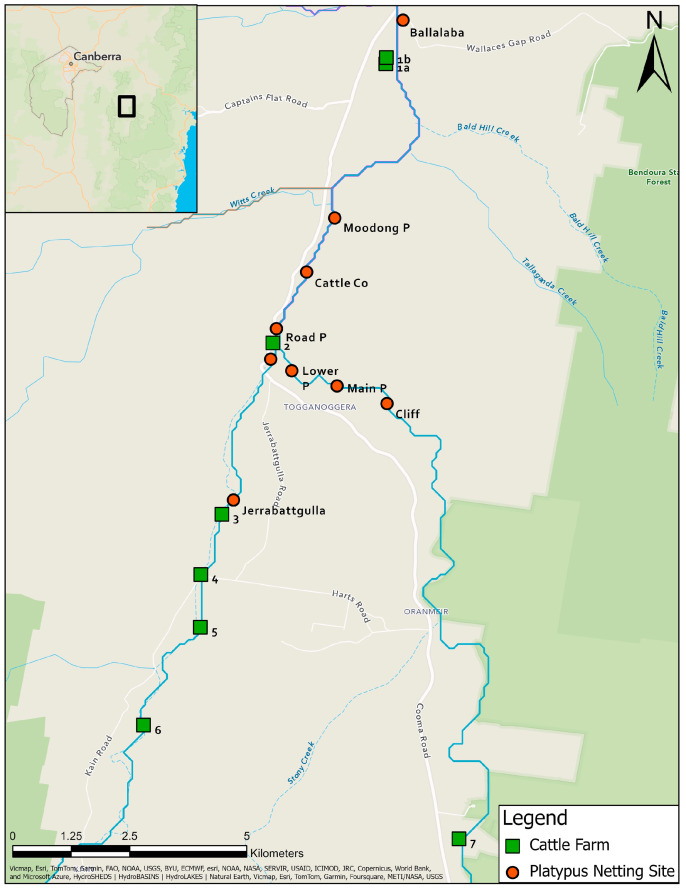
Locations of platypus and cattle sampling sites in the study area along the upper Shoalhaven River and Jerrabattgulla Creek, NSW. Named sites with round symbols are those where platypuses were captured and sampled. Numbered sites with square symbols are the locations of the cattle farms.

Eight farms between Ballalaba and Jerrabattgulla, NSW with paddocks along the upper Shoalhaven River and Jerrabattgulla Creek were identified (Figure 2). Whole-herd sampling of blood from cattle was undertaken in 1985 for the Australian Bovine Brucellosis and Tuberculosis Eradication Campaign. Blood was collected using a plain vacutainer from the tail vein and sent to RVL Glenfield, NSW. Clotted blood was centrifuged at 2000× *g* and placed at 4 °C. Mobs of cattle with direct access to the river/creek were identified and representative blood samples were re-directed for leptospiral microscopic agglutination test (MAT) testing, which was completed within a few days of collection. None of the cattle had been given a leptospiral vaccine.

### 2.8. Culture of Leptospires

Cultures were performed on homogenised kidney/urine/blood samples from necropsied animals and on whole-blood samples from live platypuses. Blood cultures from the latter were set up in the field using freshly collected blood. One drop of whole blood from the tip of the hypodermic collection needle was inoculated into two 15 cm glass tissue culture tubes containing 2.7 to 4 mL of liquid EMJH medium (Leptospira base medium EMJH code 0794 with Leptospira enrichment code 0795, Difco-Bacto, Mt Pritchard, Australia). The medium was similarly inoculated with kidney homogenate or urine. Cultures were performed at RVL Wagga Wagga, incubation was aerobic at 30 °C for 20–26 weeks, and tubes were examined weekly via dark-field microscopy along with appropriate control leptospiral cultures.

### 2.9. Microscopic Agglutination Test

Serum samples were tested soon after collection and/or after archiving. Prior studies have revealed that *L. interrogans* serovar Hardjo is the serovar of greatest relevance for the platypus [9,11,25]; information on other serovars is available in prior reports and was not evaluated further in this study. Sera from platypuses collected in 1981 and 1982 (n = 17) were tested at the Veterinary Research Institute Parkville, VIC at a screening dilution of 1:32 [25]. Some sera from platypuses (n = 7) collected in 1985–1986 and all sera from cattle collected in 1985 were tested at the RVL Glenfield, NSW at a screening dilution of 1:100. Sera collected in 2001 (n = 22) were tested at Queensland Health Scientific Services at a screening dilution of 1:50 [25]. Sera collected in 2009 from NSW (n = 26) were tested at Mount Pleasant Laboratories, Tasmania at a screening dilution of 1:100. Samples in the Platypus Serum Bank (n = 392) were tested in 2023 at Elizabeth Macarthur Agricultural Institute, NSW (EMAI) at a screening dilution of 1:50. The MAT was performed according to methods adapted in each laboratory as generally described [40,41]. Samples that did not agglutinate at the screening dilution were defined as negative, while those with agglutination at this or a higher dilution were defined as positive.

### 2.10. Statistical Analysis

#### 2.10.1. Serology

The correlation between prior (soon after collection) and newly obtained (2023) MAT titres was determined using Spearman’s rank correlation test with a two tailed *p* value (Prism Ver 10, Graphpad, Boston, MA, USA), while test sensitivity was compared using McNemar’s test (Genstat Ver 22, VSN International, Hemel Hempstead, UK).

Apparent seroprevalence was defined as the percentage of MAT-positive serum samples in each category (location, age, sex, year, lactation status) because (i) most animals (92.9%) were sampled only once and (ii) the inter-sampling interval for 29 platypuses sampled more than once was usually more than one year (see results) and the dichotomous outcome for some animals varied between positive and negative over time. The 95% binomial confidence limits for the proportions were determined (Genstat). Seroprevalence between locations was compared using a two-sample binomial test (Genstat). Contingency tables were prepared; factors associated with MAT status (age, sex, lactation status) were identified using a chi-sq or Fisher’s exact test (Prism). Data on lactation status were censored so as to include animals tested for lactation only in November, December, January and February, because lactation outside that period was extremely unlikely based on 27 years of data from the upper Shoalhaven River population [37,38].

#### 2.10.2. Spatial Analysis

Each point location represented an individual serum sampling event. The point locations were geographically recorded in decimal degrees and imported into a Geographical Information System (GIS) using World Geodetic System (WGS) 84. The spatial data were transformed into GDA2020/MGA zone 55. Point locations that overlapped due to multiple samplings at the same study site were randomly dispersed within a 10 m radius of the original location using the ‘Disperse Markers’ tool (ArcGIS Pro ver 3.1, Environmental Systems Research Institute, Brisbane, Australia). All spatial analyses were undertaken using ArcGIS Pro 3.1.

Hot spot analysis of platypus transmission locations was undertaken after aggregating data by state: ACT and NSW combined due to proximity, VIC, and TAS. Data from the Wollondilly River were not included in the NSW analysis due to a lack of information about sampling location. A hot spot analysis (Getis-Ord Gi*) [42] was performed separately for each state to determine the spatial association between platypus leptospirosis cases. The output was a new dataset characterised by the Getis-Ord Gi* statistic expressed as a z-score for the point location of each serum sample that determines if it clusters spatially relative to other point locations throughout the study area [42]. The Getis-Ord Gi* statistic considers the proximity of each point location to all other point locations in addition to whether the serum sample was positive or negative [42]. Statistically significant positive z-scores deemed a point location to be a hot spot, while significant negative z-scores deemed a point location to be a cold spot [42]. Positive point locations surrounded by other positive point locations were deemed hot spots and negative point locations surrounded by other negative point locations were deemed cold spots, relative to the entire population. A negative point location could be deemed a hot spot if surrounded by a greater number of positive point locations, while a positive point location in proximity to a greater number of negative point locations could be deemed a cold spot. Each hot spot and cold spot was classified by its significance level at the 90% (*a* < 0.10), 95% (*a* < 0.05), and 99% (*a* < 0.01) confidence intervals. A cluster is a group of features that appear closer together than the overall random distribution. A region (sub-catchment of a drainage division) in which a high-density cluster of hot spots occurs can be considered an area of high risk for leptospirosis transmission and vice versa.

A bivariate dataset was created to analyse possible transmission locations of leptospirosis between cattle and platypuses using a colocation analysis [43]. Only cattle and platypus serum samples that returned a positive MAT result in the upper Shoalhaven River in NSW (Figure 2) were included. For cattle samples, the paddock closest to the Shoalhaven River where cattle were able to interact with the water was used as the point location. The output of the analysis is a new dataset characterised by the colocation quotient (LCLQ) [43]. LCLQ values greater than 1 represent colocation between the two features [43], meaning that the proportion of platypus (feature A) within a cluster of cattle (feature B) is greater than the global proportion of platypus (feature A). That is, if a pair comprising one cattle and one platypus point location are within closer proximity relative to the entire population of cattle or platypus point locations, the point locations will be deemed to be co-located. LCLQ values less than 1 represent isolated study sites [43], meaning that there was an uneven distribution of either positive cattle or platypus point locations or the point location was spatially segregated from other point locations. For instance, a platypus point location (category A) that is located a great distance from other platypus or cattle point locations, with a low number of surrounding similar platypus point locations (category A), and even fewer surrounding cattle point locations (category B), relative to the entire population, will be deemed isolated. Non-significant co-location or isolation was reported when the point locations had *p* values close to significance (near miss). The colocation analysis was performed twice, firstly with cattle point locations as the category of interest (category A) and secondly with platypus point locations as the category of interest (category A).

## 3. Results

### 3.1. Validation of Testing of Archival Samples

**Figure 3:**
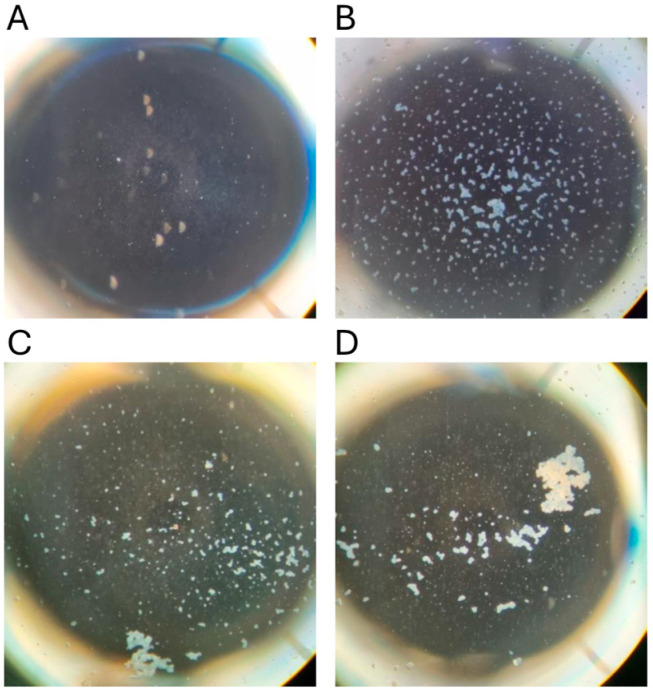
Microscopic agglutination test reactions conducted in 2023. Dark-field photomicrographs of representative microtiter plate wells. (**A**) Negative control (no serum) with no agglutination; (**B**) Fresh bovine serum (dilution 1:200); (**C**) Platypus serum #22 stored since 1985 (dilution 1:1600); (**D**) Platypus serum #397 stored since 2012 (dilution 1:3200).

To confirm the suitability of archival sera for the MAT, 107 samples with results from tests conducted between 1985 and 1987 were retested in 2023. The agglutination reactions were typical of those obtained using freshly collected mammalian sera, displaying classic dense white agglutinated clumps of leptospires forming the typical lace network of *L. interrogans* serovar Hardjo (Figure 3). There was significant correlation of the titres obtained from these sera in 2023 with their prior titres (r = 0.82, *p* < 0.0001). Using dichotomous classification, 73 were positive in 2023 (screening dilution 1:50) compared to 53 in 1985–1987 (screening dilution 1:100). The apparent sensitivity of the MAT conducted in 2023 was higher (Chi sq 3.72, *p* = 0.027). A prozone effect [44] was observed in one sample but not above the 1:200 dilution. Therefore, all archival sera were subsequently screened at dilutions of 1:50, 1:100 and 1:200, but no further examples of the prozone effect were observed.

### 3.2. Seroprevalence of Leptospiral Infection in Platypuses

The locations of sample collection from platypuses in each region are shown in Figure 1, while detailed locations for the upper Shoalhaven River are presented in Figure 2.

Results from 464 serum samples were available for analysis (Table S1). These comprised 392 archival samples tested in 2023 and 72 samples which were not represented in the serum bank but for which there were prior results (45 of which were positive): the upper Shoalhaven River NSW collected in 1981–1982 (n = 17) [11] or 1985–1986 (n = 3); the Murrumbidgee drainage division collected in 1986 (n = 3) or 2009 (n = 26); the Lachlan River collected in 1985 (n = 1); and the Wollondilly River collected in 2001 (n = 22) [25]. Among the archival samples were 45 from TAS that had been tested previously [9], but the remainder had either not been tested or the MAT results obtained near the time of collection had not been reported (n = 107).

The 464 serum samples were from 411 individual platypuses sampled between 1981 and 2012 from NSW, ACT, VIC, and TAS (Table 1, Figure 1); 29 platypuses had been sampled more than once. Most of the samples were collected from NSW and TAS, but there were 26 from VIC and seven from the ACT. In NSW, four river basins were sampled, and one, the Murrumbidgee, was contiguous with the ACT. Three river basins were sampled in VIC and seven in TAS (Table 1, Figure 1). The majority of platypuses were free-living, but five samples were collected from captive animals (n = 4); these are shown separately in Table 1 rather than being associated with a river basin due to uncertainty about where they had been captured or where they had been exposed to leptospires.

**Figure 4:**
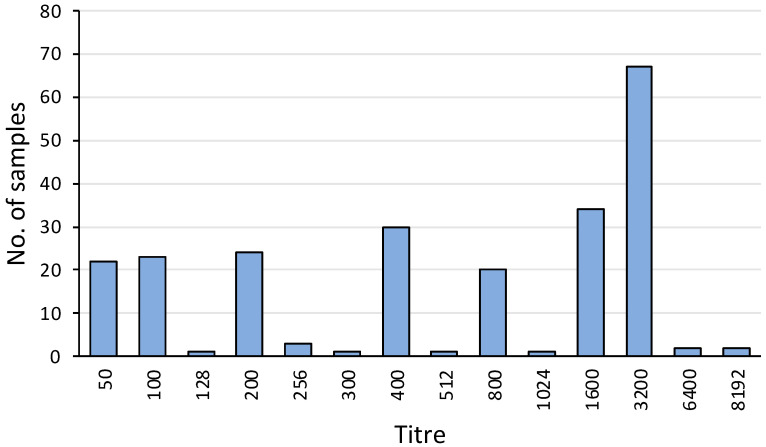
Frequency distribution of MAT titres against *L. interrogans* serovar Hardjo among the 231 platypus sera with agglutination activity at or above the screening dilution. Of these positive samples, 186 were from tests conducted in 2023 and 45 were from tests conducted in 1981, 1982, 1985, 2001, and 2009. Data include 22 positive samples from published reports [11,25].

Overall, 231 out of 464 (50%) serum samples were positive for agglutinating antibody titres against *L. interrogans* serovar Hardjo. The titres ranged from 50 to 8192, with a mode of 3200 (Figure 4). The apparent seroprevalence was significantly lower in TAS (23%) compared to NSW (67%) (*p*< 0.001) and VIC (54%) (*p* < 0.01) (Table 1).

In NSW, most samples were from the upper Shoalhaven River (seroprevalence 67%) (Figure 2), but there were 26 samples from the Murrumbidgee River basin and 22 from the Hawkesbury River basin, where the seroprevalence was also high. The majority of samples from VIC were from the Goulburn River basin (seroprevalence 73%) and the Yarra River basin (50%). The highest seroprevalences recorded in TAS were from the Tamar River basin (32%), followed by the Derwent River basin (25%). There was a relatively high number of samples from the Smithton–Burnie Coast basin (n = 59), but the seroprevalence there was comparatively low (15%) (Table 1).

### 3.3. Platypus Hot-Spot Analysis

**Figure 5:**
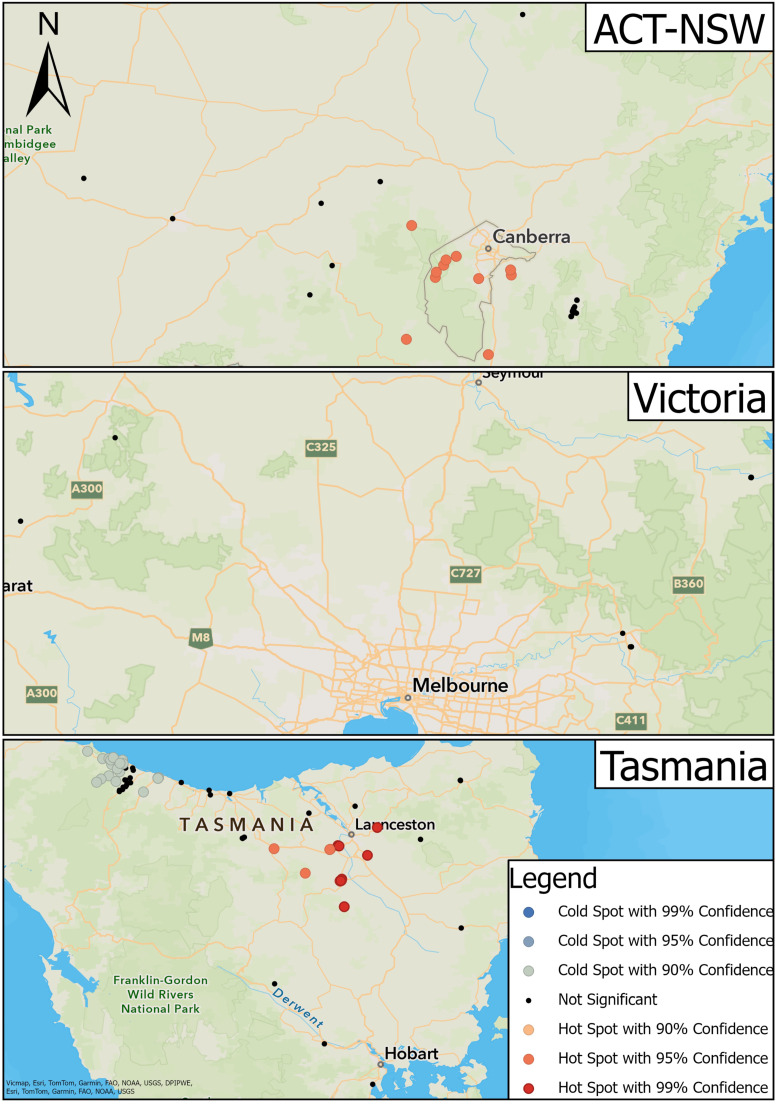
Results of hot spot analysis for the transmission of leptospirosis in platypuses.

There was a cluster of hot spots in ACT-NSW located throughout the ACT and the surrounding regions (95% confidence) (Figure 5). Point locations in the more distant surrounding regions such as the upper Shoalhaven River, Wagga Wagga, and Tumut, NSW were not significant (Figure 5). Sixteen hot spots comprised MAT-positive serum samples, while four comprised MAT-negative serum samples. The surrounding non-significant point locations had a mixture of positive and negative serum samples.

There was greater variation in hot and cold spots in TAS (Figure 5). Hot spots were located towards the midsection of the study area between Deloraine and the surrounding regions of Launceston and Longford (90% and 95% confidence) (Figure 5). Of these, 26 point locations displayed positive serum samples while 52 displayed negative serum samples. In the latter, the concentration of positive samples was greater than negative samples compared to the surrounding regions. Cold spots were located to the northwest of the state around Boat Harbour and Burnie (90% confidence); 29 displayed negative serum samples and seven displayed positive serum samples. A cluster of non-significant point locations was situated between the cold spot clusters at Boat Harbour and Burnie.

There were no instances of hot or cold spots in VIC (Figure 5).

### 3.4. Age

**Figure 6:**
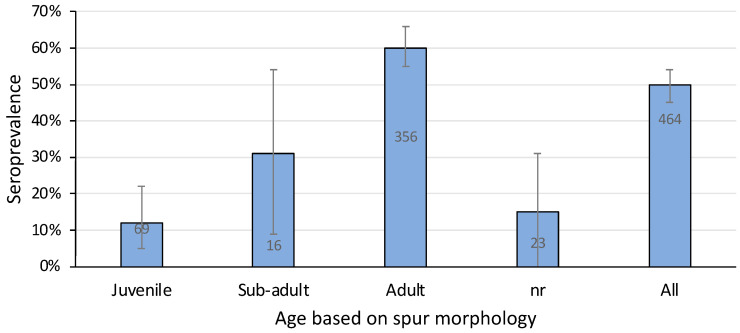
Seroprevalence of *L. interrogans* serovar Hardjo in platypuses according to their age using data pooled from all sampling sites. nr, not recorded. Bars show prevalence with 95% confidence intervals and the number of samples tested. Data include 39 samples from published reports [11,25].

A total of eight out of 69 juvenile platypuses (12%) and 215 out of 356 adults (60%) were positive for agglutinating antibody titres against *L. interrogans* serovar Hardjo (Figure 6). Sub-adults, all 16 of which were male because it is not possible to define sub-adult females based on spur morphology [32,33], had an intermediate seroprevalence (31%) (Figure 6). Adult platypuses were significantly more likely than younger animals to be positive (Chi-sq 17.0, *p* = 0.0002)—that is, the chance of leptospiral infection increased with age.

### 3.5. Sex

The proportion of females among platypuses sampled from TAS was significantly lower than from NSW (Chi sq 12.5, *p* = 0.0004), and therefore a comparison of seroprevalence between female and male platypuses was carried out at a regional level. There was no significant difference in seroprevalence between males and females in NSW, TAS, or VIC (Table 2).

### 3.6. Seroprevalence over Time in the Upper Shoalhaven River

**Figure 7:**
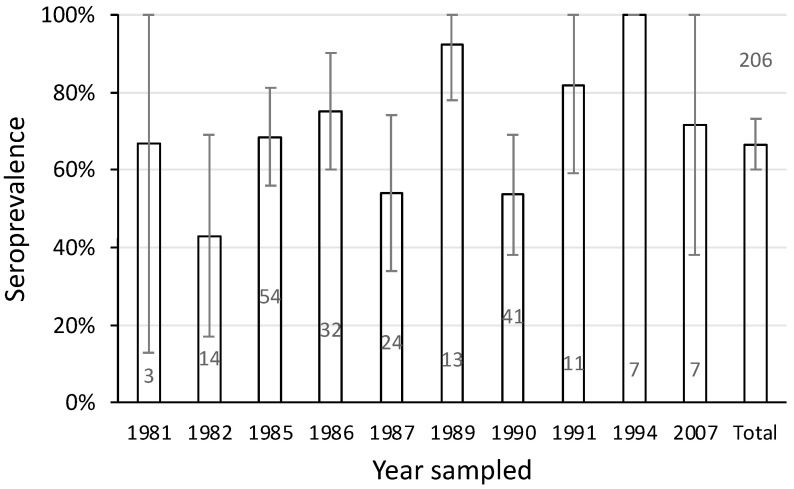
Seroprevalence of *L. interrogans* serovar Hardjo in 206 samples from platypuses in the upper Shoalhaven River sampled between 1981 and 2007. Bars show the seroprevalence with 95% confidence limits and the number of samples tested. Data include 17 samples from a published report [11].

The seroprevalence of leptospirosis in platypuses varied over the years between 1981 and 2007. The minimum level was 43% in 1982 (Figure 7). While sample size and therefore the confidence of the estimate was low in some years, high seroprevalence was nevertheless recorded in some years where sample size was large. For example, in 1985 the seroprevalence was 69% among 54 samples (Figure 7). Overall, the pattern of antibody response in the upper Shoalhaven River population was consistent with leptospiral exposure spanning 27 years.

### 3.7. Repeated Sampling of Individual Platypuses over Time

#### 3.7.1. Shoalhaven River

Twenty-seven platypuses (21 female, six male) from the upper Shoalhaven River were sampled more than once between 1985 and 1992 (Table 3). Most were adults when first sampled, although there were two juvenile females, one juvenile male, and two sub-adult males. Individuals were mostly caught at the same location each time (Figure 2)—Ballalaba (n = 1), Main Pool (n = 17), Lower Pool (n = 7), or Jerrabattgulla (n = 2)—but four animals from Main Pool or Lower Pool, which are separated only by a long riffle series, were caught on occasion in the other pool.

**Figure 8:**
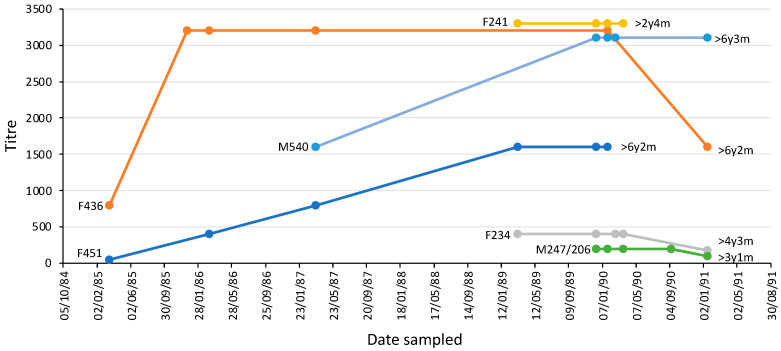
*L. interrogans* serovar Hardjo MAT titres over time in six individual platypuses from the upper Shoalhaven River that were sampled at least four times. Each platypus is shown using a different coloured line; the markers indicate the times of sample collection. The sex and identification number of each platypus and its age when last sampled are shown. F, female, M, male. The data for animals with identical titres have been offset to enable visualization.

The majority of platypuses (n = 20) had an MAT titre > 50 on each occasion, spanning intervals of one month to 70 months (average 21 months) (Table 3). The others had a titre that resolved over time from positive to <50 (n = 2) or never had a titre (always <50) (n = 5). Two animals (one male, one female) were sampled six times over five or six years, three (one male; two female) were sampled five times, while one (female) was sampled four times (Figure 8). Female platypus 451 and male platypus 436 were more than six years old when last sampled and had been seropositive over most of their lives until that point.

#### 3.7.2. Wollondilly River

One adult male platypus was sampled twice, 14 days apart in 2001. The MAT titre increased from 100 to 200 over this time [25].

### 3.8. Lactation

Concurrent assessments of MAT titre and lactation status were conducted on 24 individual platypuses during breeding seasons between 1985 and 1994. Three of these platypuses were assessed in more than one breeding season. All of the lactating females were MAT-positive, as were 88% of the non-lactating females. There was no significant effect on lactation status attributable to MAT status (*p* > 0.05) (Table 4). Eight of the 15 seropositive and one of the two seronegative non-lactating platypuses were found to be lactating in a later breeding season.

### 3.9. Culture of Leptospires

No isolates of leptospires were obtained from any of the 86 blood samples collected from live platypuses in 1985 and 1986, nor from kidney, urine, or blood samples from four platypuses that drowned in fishing nets (Table 5 and Table 6).

### 3.10. Histopathology

**Figure 9:**
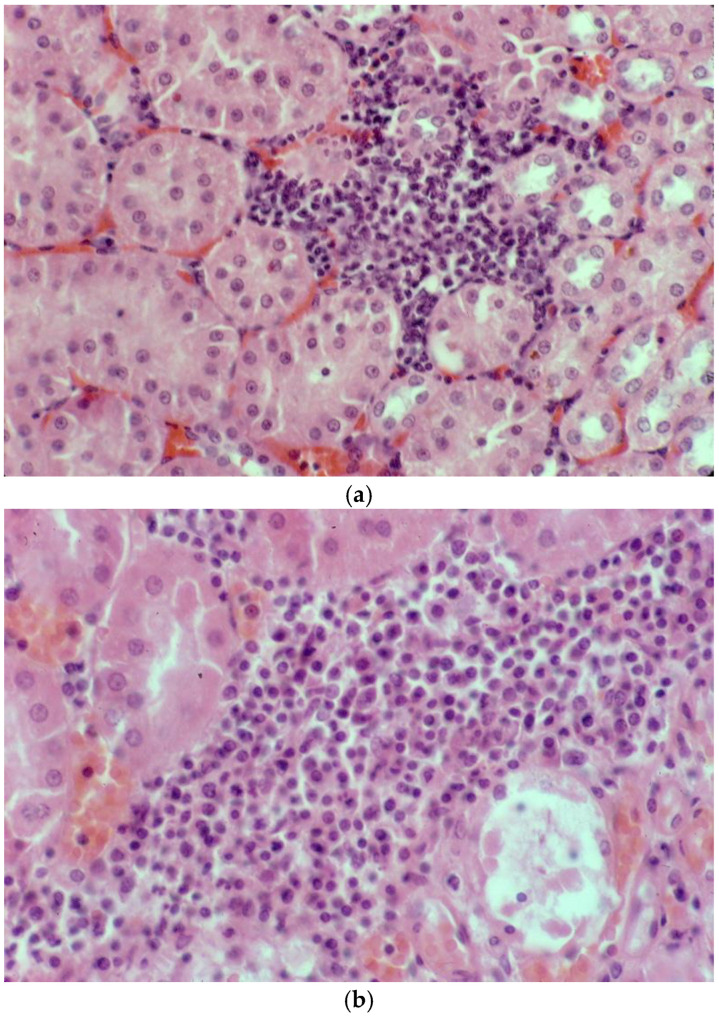

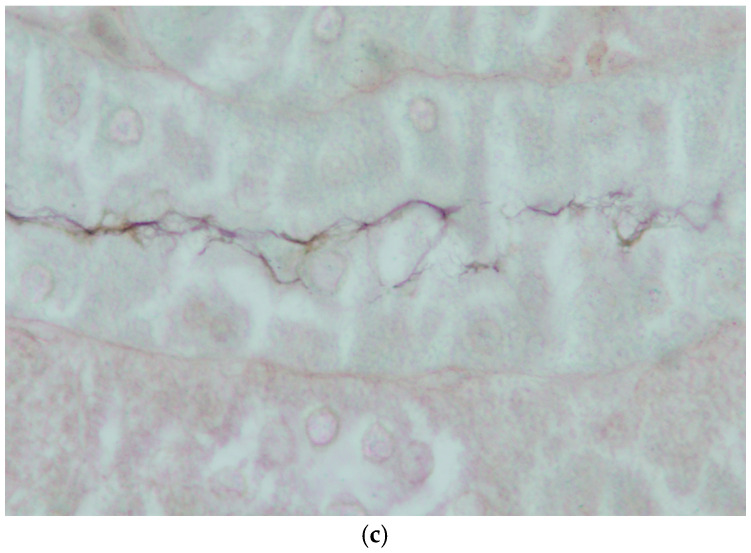
Histopathology of the kidney of platypuses. (**a**) Focal interstitial infiltration of lymphocytes in the renal cortex in platypus RN86/112-1 (H&E); *L. interrogans* serovar Hardjo MAT 100. (**b**) Focal interstitial infiltration of lymphocytes and plasma cells and necrosis of the adjacent renal tubular epithelium in platypus RN86/112-2 (H&E) *L. interrogans* serovar Hardjo MAT 300. (**c**) Abundant leptospires within a renal tubule of platypus RN86/112-2 (oil immersion, Warthin Starry) *L. interrogans* serovar Hardjo MAT 300.

Mild non-suppurative focal to multifocal interstitial nephritis was present in the renal cortex of three of four platypuses that drowned in fishing nets in NSW (Table 5). The cellular infiltrates were composed mainly of lymphocytes with variable numbers of plasma cells (Figure 9a,b). In one of these platypuses, the epithelium of occasional renal tubules lying adjacent to cellular infiltrates was necrotic (Figure 9b). Large numbers of leptospires were observed within the renal cortical tubules of all three platypuses in silver-stained sections (Figure 9c). All three platypuses had MAT titres against *L. interrogans* serovar Hardjo (Table 5). The fourth platypus did not have interstitial nephritis, there were no visible leptospires in the silver stains, and it was negative in the MAT. Three platypuses had viral inclusion bodies in the tubular epithelial cells in the renal medulla [45] that were not associated with cellular infiltrates in the renal cortex.

### 3.11. Seroprevalence of Leptospiral Infection in Sympatric Cattle

**Figure 10:**
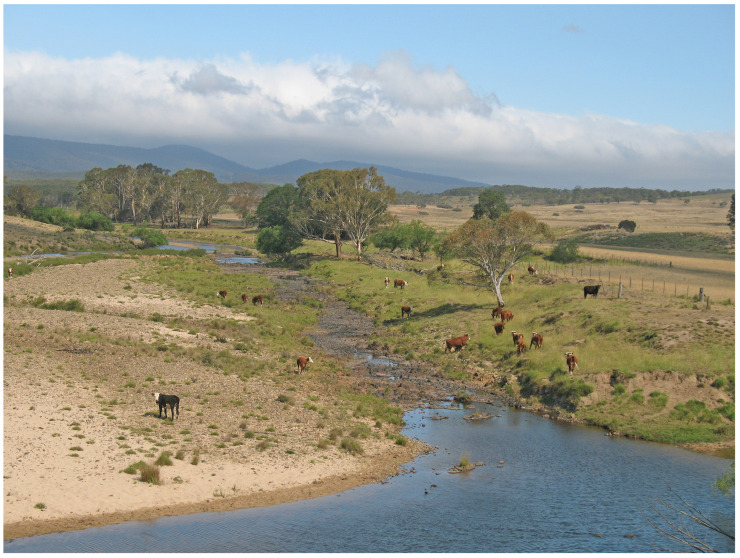
Cattle had direct access to the unfenced reaches of the river or creek in the upper Shoalhaven River study area. Dry riffle area between the Lower and Junction pools (Figure 2) during a period of low rainfall.

Titres against *L. interrogans* serovar Hardjo were detected in cattle from six of ten herds sampled on eight farms along the upper Shoalhaven River in 1985 (Table 7 and Table S2, Figure 2). All of the cattle had direct access to the upper Shoalhaven River or Jerrabattgulla Creek to obtain drinking water (Figure 10). Four of the farms were located immediately adjacent to river pools in which platypuses were sampled, but four farms were upstream (Figure 2), and of those, two had cattle with *L. interrogans* serovar Hardjo titres. Two of the authors reported seeing cattle urinating directly into or immediately adjacent to the river. Among the herds with titres, the apparent seroprevalence ranged from 1.3 to 40%. The highest titre was 100 in three herds, while most positive cattle had a titre of at least 100, with maximum titres of 300 in two herds and 2700 in another.

### 3.12. Platypus and Cattle Colocation Analysis

**Figure 11:**
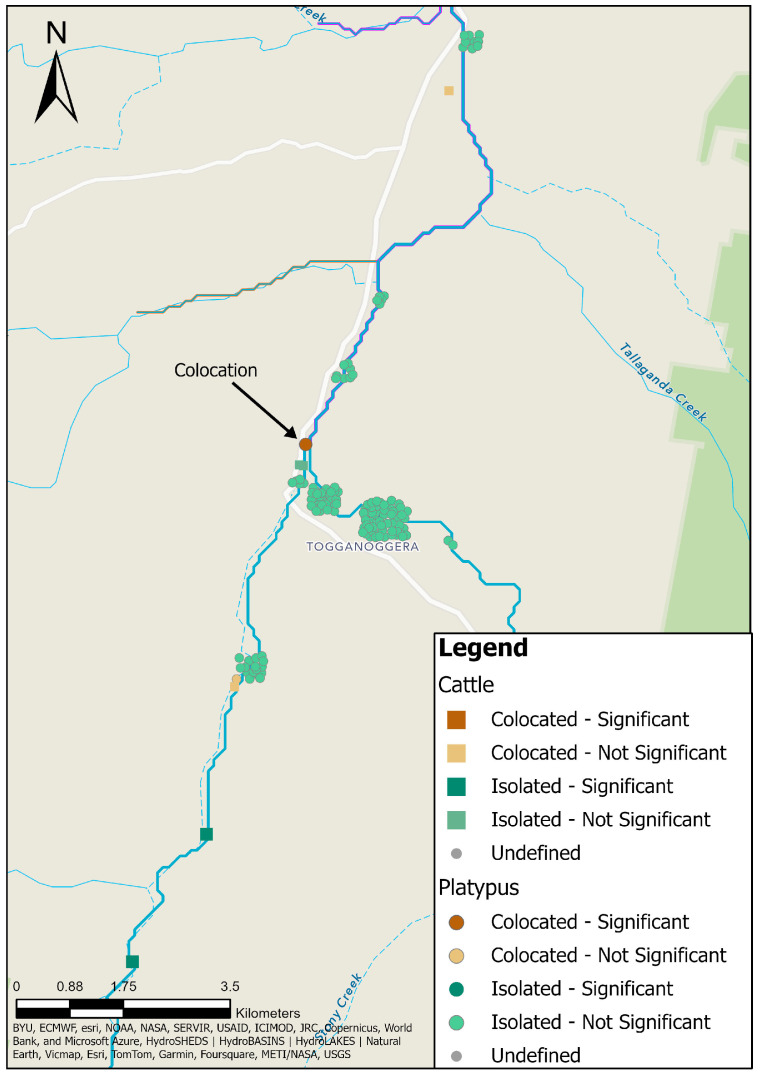
Results of colocation analysis using platypuses (round symbols) or cattle (square symbols) as the category of interest. There was one significant colocation in the centre of the study area.

The colocation analysis was performed twice, to accommodate cattle or platypuses as the category of interest, and the results of both analyses are displayed in Figure 11. There was one instance of significant colocation, but only when the category of interest was platypuses, signifying the possible transmission of leptospirosis between platypuses and cattle. The colocation occurred between a single 1985 MAT-positive platypus point location at Road Pool and two 1985 MAT-positive cattle point locations at Site 2 (Figure 11). One platypus point location and two cattle point locations were determined to be co-located, but not significant (Figure 11).

There were two significant isolated point locations (Figure 11), but only in the analysis in which cattle was the category of interest. These were due to the absence of any serum samples from platypuses in proximity to the cattle at these locations, along with a low number of similar cattle point locations to the far south of the study area. Clustering of non-significant isolated point locations occurred in the north of the study area in Ballalaba and to the north of Jerrabattgulla (Figure 11).

No study site was deemed as undefined.

### 3.13. Captive Platypuses

Four captive platypuses were sampled in this study, one of which provided two serum samples. Two animals had positive MAT results for *L. interrogans* serovar Hardjo. The platypus from NSW had a titre of 3200. The other, a juvenile female from VIC, was seronegative (titre < 50) when first sampled in 1991, two months after entering captivity. This individual had a titre of 1600 when tested 17 months later (Table 5), but the location of exposure was uncertain.

## 4. Discussion

### 4.1. Overall Findings

*L. interrogans* serovar Hardjo infections were widespread and common in platypus populations in southeastern Australia, with an overall seroprevalence of 50%. The affected populations are in rivers flowing both east and west from the Great Dividing Range on mainland Australia and flowing both north and south from the central highlands of TAS. Populations on either side of these watersheds are geographically separate. Platypus populations in QLD and in SA, where the species is rare, were not assessed due to a lack of testing. One platypus in QLD was tested for leptospiral infection in 1964 by Emanuel et al. [46], who obtained a negative result.

In the upper Shoalhaven River, NSW, there was evidence of ongoing antigenic stimulation from leptospires over 27 years. This involved more than half the population, with individual animals being seropositive for *L. interrogans* serovar Hardjo for at least six years. This suggests an ongoing, stable, endemic leptospiral infection.

In TAS, the seroprevalence of *L. interrogans* serovar Hardjo in seven river basins was lower than in the drainages on the mainland, confirming a prior comparison made between the Smithton–Burnie Coast river basin (Inglis River catchment) and the small dataset from the mainland that was available then [9].

### 4.2. Potential Transmission Locations

Hot spot analysis identified regions and local clusters in which there was a higher prevalence of MAT-positive platypuses. Hot spots occurred even in the presence of negative serum samples (Figure 5); this was apparent in the TAS analysis, where negative serum samples outnumbered positive serum samples. This was due to the higher prevalence of positive serum samples within these local clusters compared to other locations. In the TAS analysis, there were also cold spots that contained positive serum samples, because the prevalence of negative serum samples was higher within these clusters than elsewhere. It is likely that the hot spots and cold spots represent areas of high and low leptospirosis transmission, respectively. Similar spatial relationships were found among dogs with leptospirosis in the United States [47]. These authors noted that while these areas may represent potential leptospirosis transmission zones, specific wildlife reservoir factors could be involved [47]. Likewise, the inclusion of environmental risk or transmission factors may further explain areas of high transmission for platypuses. However, there is a possibility that the identification of hot spots could be due to the repeated sampling of the same point locations, inherently creating clusters at each sampling location. This problem could be overcome in future by means of systematic random sampling of a grid overlaid on the area, as was performed to select areas for sampling rodents for leptospirosis [48]. This was not possible in our study because the majority of samples were from archived sets which had been collected from multiple studies over time.

### 4.3. Age

The age of platypuses is determined by means of examination of a keratinous spur on the hind leg. In juvenile females, a vestigial spur sheath is present until the end of the first year of life, after which it is impossible to determine age with any more certainty. In males, which have a well-developed spur, three categories correspond to juveniles in their first year of life; sub-adults in their second year; and adults more than two years of age [33]. The seroprevalence of *L. interrogans* serovar Hardjo was lower in juvenile than adult platypuses of both sexes. In males, there was a progressive increase in seroprevalence from juveniles to sub-adults to adults. There are several possible explanations for this observation, including juvenile mortality [9,25], incremental environmental exposure, or passive immunity. There could have been an epidemic (leptospirosis or another condition) that killed juveniles in preference over adults so that there were fewer surviving juveniles with titres than adults with titres. However, juvenile platypuses with titres against *L. interrogans* serovar Hardjo were recruited into the population and there has been no reported clinical evidence of morbidity or mortality associated with leptospirosis in platypuses, either in captivity or in the wild anywhere across their distribution. Accumulation of infections over time is most likely due to exposure to leptospires in water. Nesting platypuses spend the first months of their lives entirely in riparian burrows away from potentially contaminated river water [1,38], and despite direct contact with adult females that may be carrying and shedding leptospires, they mostly evade the infection until later in life. Leptospiral infection is not transmitted efficiently to them by direct contact with adult females in the burrow. In common brush-tailed possums in both Australia [49] and New Zealand [50], the prevalence of *L. interrogans* serovar Balcanica increased progressively with age. This was partly attributed to the protection of juveniles by maternal antibodies (colostral immunity) [49]. This has also been proposed as a reason for the increasing prevalence of *L. interrogans* serovar Hardjo after the first few months of life in sheep [51]. Passive immunity might also protect juvenile platypuses from infection with leptospires during the early stages of neonatal life in the burrow.

### 4.4. Sex

There were no sex-based differences in seroprevalence in platypuses sampled from NSW, TAS, or VIC, although in the small sample from the Wollondilly River, NSW, male platypuses had a higher seroprevalence than females [25]. A sex effect is difficult to explain in terms of differences in potential exposure to leptospires because male and female platypuses frequent the same aquatic environments, while transmission could occur between the sexes through direct contact during courtship and copulation [52] and might also occur in riparian resting burrows. Platypuses use a number of resting burrows, including the use of the same burrow by both males and females at different times [53]. Some radio-tracked individuals have been found to use the same burrow or burrow complex simultaneously; however, to date there are no reports of an adult male and an adult female occupying a burrow at the same time [53].

### 4.5. Impact of Leptospirosis on Platypuses

Notwithstanding the difficulties related to the clinical assessment of health status in free-ranging wildlife species in general, and in cryptic species like the platypus in particular, the authors did not observe any clinical signs of disease that could be attributed to leptospiral infection in the upper Shoalhaven River population. This has been the subject of intensive ecological study over the past 40 years [54]. Similarly, clinical leptospirosis has not been reported in captive platypuses or among the rescued platypuses that have been presented to zoos and wildlife centres for veterinary care [36].

In the four platypuses that were made available for necropsy, the impacts of leptospiral infection were very mild, based on histopathological lesions in the kidney (Table 2, Figure 9). Similarly, mild chronic interstitial nephritis was present in five out of 20 captive platypuses in VIC [55]. Such lesions would not impact renal function.

Being seropositive did not affect the likelihood of lactation in platypuses in the upper Shoalhaven River population. There was a high seroprevalence of *L. interrogans* serovar Hardjo in both lactating and non-lactating platypuses, and seropositive platypuses were just as likely as seronegative platypuses to be lactating in subsequent years. There was evidence from the lactation studies and from the appearance of juveniles in the river after each breeding season, that females in the upper Shoalhaven River population produced young each year despite endemic leptospirosis [38,56]. Juvenile recruitment to this population continued even during periods of severe drought [38], and population studies reported the upper Shoalhaven River population to be stable and not in decline [54,57]. These findings do not support the suggestion that females with leptospirosis may not be able to nurse their young [25] or are reproductively unsuccessful.

### 4.6. Effects of Quality and Consistency of MAT Data

The MAT is a complicated, labour-intensive test that requires specialist skills to undertake, interpret, and control [58]. Titres could be affected by many factors including the culture medium used to propagate the live antigen, the specific strain of leptospire used as the antigen, the passage level of the leptospiral culture, the duration of incubation of the culture, the density of the leptospiral suspension, and operator effects such as the interpretation of the endpoint under dark-field microscopy. This can lead to variation between and within laboratories. Therefore, it was considered important to have data from a single run of tests performed by one experienced operator in one laboratory; 85% of the MAT results used in the analyses were obtained in this way in 2023. Nevertheless, paired results obtained from sera tested more than 35 years apart in different laboratories were well correlated. For prevalence estimates, samples were dichotomised as positive–negative using a screening dilution of 1:50 in 2023 and 1:32 (n = 17 samples), 1:50 (n = 22), or 1:100 (n = 33) for historical data. The tests using a screening dilution of 1:100 may have lower sensitivity, all other things being equal [19], but as these represented only 7.1% of the samples, impacts on the analyses would be minor.

### 4.7. Platypus Serum Bank

The preservation of the activity of agglutinating antibodies in platypus serum samples that had been stored at −20 °C since the 1980s was consistent with data on the robust nature of human immunoglobulins in the Janus Serum Bank in Norway after 25 years of storage [59]. The agglutination reactions observed under dark-field microscopy were typical of those obtained with freshly collected sera. The value of archived serum samples for sero-epidemiological studies should be recognised more widely given the costs and ethical and welfare issues associated with research on protected wildlife.

### 4.8. Biological Meaning of MAT Titres

The MAT remains the standard test for the detection of antibodies against leptospires in the serum of animals and humans. It has high sensitivity and specificity, provides reliable evidence of infection for clinical diagnosis, and provides epidemiologically useful information about leptospiral serogroups/serovars for prevalence surveys and in disease control programmes [16,58,60,61]. The bacterin vaccines deployed to control and prevent leptospirosis are described in terms of the serovars they contain [62]. Certain serovars are associated with particular maintenance hosts [58,63,64], and therefore the serovar is commonly determined for clinical cases across the animal kingdom for source attribution [22].

Most of the evidence of leptospiral infection in platypuses has come from the MAT, in which the magnitude of positive titres ranged from 50 to 8192. The detection of these antibodies in the serum indicates that there has been an infection with leptospires. While positive titres unequivocally indicate exposure to leptospires, the magnitudes are difficult to interpret. The titre used to define a “positive” MAT test is arbitrary, and while 100 is often used, lower titres are also consistent with prior exposure [60]. Maintenance of leptospires through chronic renal infection and urinary shedding can occur with titres of less than 100 [19,60]. A titre of 50 has been proposed as the cut-off indicating leptospiral exposure in wildlife [65].

In the context of clinical leptospirosis in newly infected animals and people, antibodies appear in the serum between 3 and 10 days after the development of clinical signs [19,58,66]. The rate of increase and magnitude of titres is affected by the background level of exposure in the population, because this affects immune memory [58]; however, a four-fold increase in titre over several weeks is considered to indicate recent infection [16,19,60]. There was one example of seroconversion from negative to 1600 in a captive platypus, but the 17-month timeframe was too long to be sure about recent exposure and it did not have clinical leptospirosis. The half-life of leptospiral antibodies was reported to be about 6 months in sheep [51], suggesting that persistent titres require ongoing antigen stimulation. Persistently high titres were seen in some animals in the upper Shoalhaven River, but stable lower-level titres were also recorded.

A positive titre does not directly confirm that leptospires are being shed in urine (leptospiruria) and nor does it indicate that leptospires have been cleared from the kidney. The specialist nature, cost, and long incubation periods required for leptospiral cultures have deterred investigations of urinary shedding in wildlife species, but the development of the polymerase chain reaction (PCR) has enabled the efficient detection of leptospiral DNA in the kidneys and urine to confirm active infection and leptospiruria. Consequently, studies reporting paired MAT and PCR results are becoming more common. While some authors acknowledge the limitations of both tests that affect the reliability of comparisons [67,68] and the risks of extrapolating information between species [19], there are trends that should be considered. In an 11-month survey of 142 urban Norway rats (*Rattus norvegicus*) with endemic leptospiral infections, 68% were MAT-positive and 80% were shedding leptospires in their urine [69]. There was a positive correlation between the MAT titre and the number of leptospires shed in the urine in this species [70]. Similarly, MAT titres were predictive of active leptospiral infection in racoons (*Procyon lotor*) [71] and California sea lions (*Zalophus californianus*) [72]. In sheep, *L. interrogans* serovar Hardjo titres but not Pomona titres were predictive of leptospiruria [51]. In another survey of free-living wildlife involving 309 animals from 16 species, the proportion of individuals shedding leptospires in urine (5.5%) was half of that with MAT titres [67]. Conversely, leptospiruria occurs in some individual animals without an MAT—for example, the Norway rat [69], house mouse (*Mus musculus*) [68], California sea lion [72] and cattle [60]—so serological data can underestimate the true prevalence of active leptospiral infections in a population. The degree of underestimation can be substantial; for example, 34% of 268 small mammals were PCR-positive compared to 11% of 151 being MAT-positive [73]. These data confirm for a variety of mammalian species that a proportion of animals with MAT titres will be shedding leptopsires in their urine, as will some without titres.

While there are still no published data for platypuses on the correlation between MAT titres and either renal infection or leptospiruria assessed by culture or PCR, the visual evidence of leptospiral colonisation of the renal tubules of three out of three MAT-positive platypuses confirmed that platypuses with MAT titres can have an active renal infection and therefore shed leptospires in their urine. Further research is warranted to determine the true prevalence of urinary shedding of leptospires among seropositive and seronegative platypuses. Similar confirmation studies have been recommended for other wildlife hosts to properly assess the risk of leptospiral transmission [74].

Only in well-defined epidemiological circumstances in which the leptospires have already been characterised do MAT titres provide reliable evidence of the identity of the infecting serovar because antigenic cross-reactions can occur between serovars usually within a serogroup [60]. In practical terms, while domestic cattle in Australia with MAT titres against *L. interrogans* serovar Hardjo can be assumed to be infected with *L. interrogans* serovar Hardjo, the same is not true of Australian wildlife species with titres against *L. interrogans* serovar Hardjo. Brenner et al. [75] classified 20 serovars in the Sejroe serogroup in addition to *L. interrogans* serovar Hardjo. *L. interrogans* serovar Balcanica is present in common brush-tailed possums (*Trichosurus vulpecula*) in Australia and New Zealand [50,76]. *L. interrogans* serovar Hardjo and Balcanica infections cannot be distinguished using the MAT [50,77]. Sullivan [78] reported four other serovars in the same serogroup as *L. interrogans* serovar Hardjo from the bandicoots *Perameles nasuta* and *Isodon macrourus* in Australia. It is necessary to isolate leptospires in order to assign them unambiguously to a serovar.

Efforts to obtain isolates of leptospires from platypuses by blood culture were unsuccessful, possibly because of the short bacteraemic time window [16,18,19] and low volume of blood that was cultured. However, several samples of kidney that contained visible leptospires and urine from drowned platypuses did not yield a leptospiral isolate either; the time that elapsed between death and the processing of samples at the laboratory may be relevant. Another possibility is that there is a fastidious leptospire present in platypuses. They have a body temperature of 32 °C [79], which is five to seven degrees cooler than most mammals, and it is possible that leptospires from platypuses have particular cultural requirements, including a lower optimal in vitro incubation temperature than is standard (30 °C) [60,80]. Some leptospires have unusual cultural requirements; the culture media HAN and T80/40/LH were recently reported to better support the growth of fastidious leptospires in *L*. *borgpetersenii* serovar Hardjo compared to EMJH [81], and should be evaluated in future studies of the platypus.

### 4.9. Who Is Infecting Whom?

While cattle are an acknowledged maintenance host for *L. interrogans* serovar Hardjo [82] and the platypus may be an accidental host, other epidemiological scenarios need to be considered to decide who is infecting whom.

#### 4.9.1. Exchange of Leptospiral Infection between Platypus and Cattle

The most obvious scenario is that cattle are the source of *L. interrogans* serovar Hardjo for platypuses [11]. MAT titres against *L. interrogans* serovar Hardjo were detected in both platypuses and cattle along the upper Shoalhaven River. The platypus population in the Wollondilly River was also thought to be at risk from domestic cattle [25]. Historically in Australia and elsewhere, the prevalence of *L. interrogans* serovar Hardjo infection in cattle appeared to increase from the 1960s onwards [78]. Approximately 17% of 2817 cattle tested from 406 farms were positive in a survey conducted in 1970–71 in NSW [83], and the infection was common in dairy cattle in Victoria (VIC), where around 50% of more than 30,000 serum samples were positive in one survey in 1970-75 [84] and more than 24% on some farms in 1979 in another [85]. *L. interrogans* serovar Hardjo was also widespread in cattle in TAS in 1970 [86]. The only more recent survey appears to be one conducted in 2017 using urine PCR, in which it was estimated that 10% of dairy herds in southwest Victoria, Australia were infected [87]. Cattle are translocated routinely around Australia during normal farming practices, often between farms and abattoirs via saleyards, but also between farms and over long distances [88]. Vaccination against leptospirosis is either not routine in cattle or compliance with recommended administration is poor [87]. For these reasons, *L. interrogans* serovar Hardjo infections in cattle are widely distributed, and this could lead to leptospiral contamination of many river systems.

In this scenario, both cattle and platypuses are infected with the same species of leptospire. Cattle grazing immediately adjacent to the pools where platypuses live are relevant, together with the cattle upstream, because leptospires have a long survival time in water and would move downstream with water flows. Exposure of platypuses to an infective dose would depend on the leptospiral contamination rate, which would depend on the prevalence of infection in cattle, their grazing management and access to the river, rainfall and water run-off into the river [89], the duration of survival of leptospires and their dilution rate, and the amount of time that individual platypuses spend in the river.

In a slight variation to this scenario, rather than there being one-way transmission, it might be two-way, meaning that some cattle are infected from platypuses. Recent evidence from New Zealand suggests that cattle can be exposed to leptospires circulating in wildlife populations [68].

However, there was only a weak spatial relationship between platypuses and cattle with evidence of *L. interrogans* serovar Hardjo exposure, identified as one instance of colocation in 1985, apparent only when the platypus was the category of interest and data from cattle for 1985 were combined with data for platypus across all years to increase the sample size. There were no instances of colocation otherwise. This suggests that there is only a weak possibility of leptospiral transmission in either direction between sympatric cattle and platypuses, but further investigation into transmission using molecular epidemiology is required for a more definitive conclusion. The colocation analysis did not consider leptospiral transfer from upstream cattle herds or platypuses, as only the inert point locations were compared. Analysis of hydrological flows and platypus movements that disperse leptospires could reveal that cattle herds upstream or platypuses both upstream and downstream are more important in leptospirosis transmission than the results presented here can indicate. Research on the movements of platypuses in several river catchments in NSW and VIC revealed mean linear movements of individuals of 1.3 km in the upper Shoalhaven River and up to 4.6 km in the Yarra River; however, the maximum movement recorded was 15 km (Appendix A, Table A1). The possibility of direct animal-to-animal transmission as well as indirect transmission in water when combined with the high seroprevalence in NSW and VIC, would likely contribute to leptospiral infection becoming widespread in each catchment.

#### 4.9.2. Independent Infection Cycles

In a second scenario, both cattle and platypuses are infected with different leptospiral species that manifest serologically as *L. interrogans* serovar Hardjo, but they have independent infection cycles that have stabilised and become endemic. That is, cattle are the maintenance host for *L. interrogans* serovar Hardjo, as is conventionally understood, and the platypuses are infected with a different leptospiral species with Hardjo antigens (see below). The colocation analysis results are consistent with this scenario. There need be no exchange of infection between the two hosts. This could explain the detection of leptospirosis in platypuses in the upper Cotter River in Namadgi National Park in the ACT (Table 1, Murrumbidgee drainage division), where livestock were absent. The high wall of Cotter Dam (built to 18.6 m in 1915, raised to 28.5 m by 1951, and replaced with an 83 m wall downstream by 2013) [90] on this river may prevent platypuses from migrating upstream into Namadgi National Park, with those already present there having an endemic leptospiral infection. Studies in the southwestern Indian Ocean islands revealed host- and leptospire species-specific associations, rather than a lack of host specificity, consistent with co-evolution of particular hosts and leptospires [23].

#### 4.9.3. Alternative Maintenance Hosts

In a more complicated scenario, both cattle and platypuses could be infected with leptospires derived from another host. Sheep also carry *L. interrogans* serovar Hardjo; the seroprevalence in sheep in VIC in 1978 and SA in 1998–1990 was about 40% [91,92]. Both the bare-nosed wombat (*Vombatus ursinus*) and the common brush-tailed possum are common in parts of NSW and VIC, and both species in these locations are known to be infected with leptospires that induce MAT reactions to *L. interrogans* serovar Hardjo [49,93,94]. Platypuses in the Wollondilly River with leptospiral titres could have become infected from sheep or bare-nosed wombats on the farm [25]; 9% of 23 wombats sampled there in the same year as the platypuses (2001) had titres against *L. interrogans* serovar Hardjo [93].

Infections with *L. interrogans* serovar Hardjo and *L. interrogans* serovar Balcanica cannot be distinguished in the MAT [50]. Common brush-tailed possums are the maintenance host for *L. interrogans* serovar Balcanica in both New Zealand and Australia [49]. Four populations of common brush-tailed possums in VIC had 16% to 66% prevalence of *L. interrogans* serovar Balcanica [49]. Experimentally, bare-nosed wombats and common brush-tailed possums are susceptible to infection with *L. interrogans* serovar Hardjo, and this wombat species, along with cattle and sheep, is susceptible to infection with *L. interrogans* serovar Balcanica [77,95].

The water rat (*Hydromys chrysogaster*) is widespread in southeastern Australia, shares habitats with platypuses, and its role in the maintenance of *L. interrogans* serovar Hardjo has been studied. MAT titres against *L. interrogans* serovar Hardjo were not detected in nine water rats from unspecified locations in southeastern Australia [96] or in 17 sampled from VIC [94]. Leptospires could not be isolated from water rats that developed low MAT titres after experimental infection with either *L. interrogans* serovar Hardjo or Balcanica or from captured wild water rats, leading to a conclusion that this species was unlikely to be a reservoir host for either serovar [95]. However, other leptospiral serovars have been found in water rats in QLD [46,97].

The network of leptospiral exposure and infection involving platypuses and cattle could be multispecies and complex. Surprising interactions can occur between terrestrial wildlife and livestock at the aquatic interface in Australia. An example is the transmission of liver fluke (*Fasciola hepatica*) from cattle to the common brush-tailed possum, which requires both species to frequent the habitat of an aquatic snail that is the intermediate host for the fluke [98].

### 4.10. Lower Prevalence of Leptospirosis in Tasmania

Considering the scenario of an alternative maintenance host, its absence could explain the low prevalence of leptospirosis in platypuses in TAS. *L. interrogans* serovar Hardjo was very rare among common brush-tailed possums and bare-nosed wombats when surveys were last conducted in TAS: one out of 57 wombats [94,99,100,101] and none of 133 common brush-tailed possums were positive for *L. interrogans* serovar Hardjo [94,100]. It is possible that the low seroprevalence in these species explains or is correlated with the low seroprevalence of leptospiral antibodies in platypuses in TAS.

It is also possible that the environment in TAS may not be conducive to the transmission of leptospires to platypuses. Factors such as water temperature and chemistry could be involved. The water temperature at the upper Shoalhaven River study site, NSW ranged from 4 °C to 28 °C between winter and summer, while air temperatures ranged from −5 °C to 33.5 °C [102,103], suggesting a wide thermal tolerance, and making temperature an unlikely factor in leptospiral prevalence in platypuses.

### 4.11. Confirming Transmission between Host Species Requires the Genomic Identification of Leptospires

The taxonomy of leptospires has been revised recently based on analysis of whole-genome sequences; there are more than 60 species grouped into four subclades (P1, P2, S1 and S2) [104]. This is a divergence from the well-accepted antigenic classification system that placed leptospires into serovars within serogroups [105]. After their discovery in the early 1900s and the later recognition of the agglutination of live leptospiral cultures by animal sera, classification was based on cross-absorption agglutination. By 2009, the pathogenic leptospires had been divided into more than 24 serogroups containing approximately 240 serovars [106]. Genomic species designations do not correspond well to the antigenic classification system because a particular serogroup/serovar can be found among more than one genomic species [75,106,107]. In the context of this study, the serogroup Sejroe which contains *L. interrogans* serovar Hardjo exists in five species defined by DNA hybridisation (*L. interrogans*, *L. weilii*, *L. santarosai*, *L. borgpetersenii*, and *L. meyeri*) [58].

There are no recent studies on the identity of leptospires in cattle in Australia, but an earlier one using cross-absorption agglutination reported that cattle in VIC were infected with *L. interrogans* serovar Hardjo [108]. Restriction endonuclease analysis of leptospiral DNA revealed that cattle in both VIC and NSW were infected with *L. interrogans* serovar Hardjo subtype Hardjobovis [109,110].

To determine whether platypuses share these infections as part of a host network or are infected with a different species of leptospire that cross-reacts with *L. interrogans* serovar Hardjo in the MAT, it will be necessary to obtain isolates and identify them using whole-genome sequencing. Purposefully collected urine samples from free-living platypuses and opportunistically collected cystic aspirates of urine and kidney from moribund/dead platypuses submitted to animal rescue services and zoos are the logical source materials. Considerable pre-planning and coordination with laboratory scientists will be necessary for such a study. Due to the lack of recent studies in cattle, the evolution of leptospiral genomic taxonomy, and occasional reports of lack of concordance of identification of leptospiral reference strains held in different reference collections [111], recent leptospiral isolates from cattle should also be evaluated. Future work should consider leptospiral cultures of domestic sheep and wildlife such as common bare-nosed wombats, common brush-tailed possums, and bandicoots to further categorise possible transmission cycles of *L. interrogans* serovar Hardjo. Studies of environmental DNA—i.e., the detection and speciation of leptospires based on DNA detection in river water and soil—would facilitate investigations of the presence of leptospires in animals occupying water bodies and adjacent land and would help to establish transmission pathways [65,112]. This would solve the mystery of who is infecting whom with leptospires in and around rivers in southeastern Australia.

### 4.12. Is the Platypus a Maintenance Host?

Establishing general criteria for a host to be defined as a reservoir of infection for a multihost pathogen is problematic and based upon ecological considerations [113,114]. There is no current definition for leptospirosis, possibly due to the paucity of individual animal longitudinal data for most free-living wildlife species. Using a laboratory mouse model, Hathway et al. [115] proposed that “a maintenance host may be defined as an animal which is capable of acting as a natural source of leptospiral infection for its own species. A maintenance population may be defined as a population of a species of animal which acts as a continuous reservoir of a serovar in a specific ecosystem”. Features of infections in maintenance hosts included high susceptibility/a low infective dose and long-term renal shedding [115]. Colonisation of the renal tubules with shedding of leptospires in urine should occur in a reasonable proportion of the population [46]. Persistent MAT titres tend to be used as a proxy for this in some species [61]. More recently, it was proposed that reservoir hosts “are uniformly recognized as asymptomatic carriers” that avoid both the disease caused by immunopathology and immunological clearance of the pathogen from the urinary system [82]. In the common brush-tailed possum, which carries *L. interrogans* serovar Balcanica, there is high seroprevalence, increasing seroprevalence with age, usually no clinical disease, mild pathology in the kidney, and an association between focal interstitial nephritis and MAT titres [49,50,94]. The lack of clinical disease with urinary shedding has been confirmed in this species following experimental inoculation with *L. interrogans* serovar Balcanica [95]. High seroprevalence (>50%) is also a feature in the racoon, which is a reservoir host [65], and the water vole (*Arvicola terrestris*), which is suspected to be one [116]

The complete absence of clinical disease is not a requirement for a species to be a reservoir host. Analysis of serological and health data from California sea lions led researchers to question whether this species is a reservoir host affected by disease when there is a lapse in herd immunity [117]. Similarly, there can be morbidity in cattle with *L. interrogans* serovar Hardjo infection [83]. The outcome of leptospiral infection depends on the degree of adaptation of leptospires to their hosts. In reservoir hosts, there is a balanced host–pathogen relationship with particular leptospires. This was illustrated in wombats: those experimentally infected with *L. interrogans* serovar Pomona died with acute leptospirosis [101], while those inoculated with *L. interrogans* serovars Hardjo or Balcanica developed MAT titres and shed leptospires in urine but did not develop clinical disease [95]. Lapses in host immunity due to concurrent infections, starvation, trauma, and other stresses theoretically could predispose to clinical leptospirosis. In this context, factors that could impact the health of platypuses have been reviewed [7,8,9,118].

The platypus has many features consistent with it being a reservoir host: high seroprevalence that is stable at the population level over many years; increasing seroprevalence with age; persistent MAT titres lasting years in individuals; mild renal pathology; the colonisation of the renal tubules by leptospires; and the probable absence of clinical disease.

### 4.13. Limitations

The seroprevalence estimates reported here are not based on random samples or a systematic survey, but rather on opportunistic sampling; therefore, it is unwise to make extrapolations to unsampled catchments within these states or to QLD or SA, where there was no sampling. However, there is no reason to think that leptospiral infection in platypuses would not be more widespread. The assessment of leptospiral transmission between hosts was constrained by a lack of direct data on long-term urinary shedding by individual platypuses, and particularly by the absence of contemporary PCR evidence—this will be a vital tool in future studies. Similarly, the lack of direct evidence of urinary shedding of leptospires by other wildlife hosts or the identification of the species of leptospires constrains definitive conclusions on transmission pathways. Understanding potential transmission due to platypus movements and the fate and transport of leptospires in watercourses also needs to be accounted for in spatial modelling. Direct, long-term observations and appropriate laboratory testing of individual platypuses in zoological collections and in translocated populations subject to intensive monitoring would enable conclusive assessment of the health effects of leptospiral infection.

## 5. Conclusions

Leptospiral infections occur in platypus populations in 14 river basins across VIC, NSW, and TAS. Seroprevalence is significantly higher in the mainland states (NSW and VIC) compared to TAS.

Seroprevalence was directly related to age and was associated with ongoing contact with the aquatic habitat. Transmission between platypuses was not due to close contact in the earth burrow. There was high seroprevalence over several decades in the upper Shoalhaven River population, and while individual platypuses had titres for up to six years, the pathogen did not affect breeding and the population appeared to be to be stable.

The role of cattle in leptospiral infection of platypuses is still not resolved four decades after it was first suggested. Furthermore, recent whole-genome analyses of leptospires mandate deeper investigation of leptospiral identity in Australia than has been conducted so far. A range of leptospiral transmission scenarios must be tested in order to understand the epidemiological connectivity between the wildlife and domestic animal hosts that have a substantial prevalence of *L. interrogans* serovar Hardjo in southeastern Australia. A landscape-wide study is recommended in which leptospires are characterised in platypuses, other wildlife species, livestock, and the soil–water environment.

## Figures and Tables

**Table 1:** Number of serum samples collected from each river basin and the prevalence of platypuses with MAT titres against *L. interrogans* serovar Hardjo. nr, not recorded.

River Basin	Years	No. Tested	No. Positive	Seroprevalence %	95% c.l. %
					
*New South Wales (NSW)*	All	256	171	67	61–73
Lachlan River	1985	1	0	0	0–98
Murrumbidgee River	1986, 1997, 2009	26	19	73	52–88
Hawkesbury River ^1^	2001	22	14	64	41–83
Shoalhaven River ^2^	1981–2007	206	137	67	60–73
Captive	1997	1	1	100	3–100
					
*Australian Capital Territory (ACT)*	All	7	6	86	42–100
Murrumbidgee River ^3^	2007, 2009	7	6	86	42–100
					
*Victoria (VIC)*	All	26	14	54	35–73
Goulburn River	1991–1992	11	8	73	39–94
Loddon River	1989–1990	2	1	5	13–98
Yarra River	1991–1992	6	3	5	12–88
Captive	1993	4	1	25	1–81
nr	nr, 1995	3	1	33	1–91
					
*Tasmania (TAS)*	All	175	40	23	17–30
Derwent River	1994–1995	4	1	25	1–81
East Coast	nr	2	0	0	0–84
Forth River	1994–1995	2	1	50	13–98
Mersey River	1994–1995	8	0	0	0–37
Piper-Ringarooma Rivers	1994–1995	11	1	09	0–41
Smithton-Burnie-Coast	nr, 1994, 2001–2012	59	9	15	7–27
Tamar River	nr, 1994–1995	87	28	32	23–43
nr	nr, 1997	2	0	0	0–84
Total		464	231	50	45–54

^1^ Wollondilly River; data from [25]. ^2^ Includes 17 samples from a published report [11]. ^3^ Cotter River; four of seven samples were collected upstream of Cotter Dam and three of these were positive.

**Table 2:** Seroprevalence of *L. interrogans* serovar Hardjo in platypuses by sex in each region. F, female; M, male; nr, not recorded.

Region	Sex	No. Sera Tested	MAT		Seroprevalence	95% c.l	
			No. Negative	No. Positive	%	%	
ACT	F	2	0	2	100	16–100	nt
	M	5	1	4	67	28–100	
NSW ^1^	F	159	50	109	69	61–76	Chi sq 0.58, *p* = 0.45
	M	97	35	62	64	54–73	
TAS	F	76	57	19	25	15–35	Chi-sq 0.17, *p* = 0.68
	M	94	73	21	22	14–31	
	nr	5	5	0	0	0–52	
VIC	F	19	8	11	58	36–80	Fishers exact, *p* > 0.99
	M	6	3	3	50	10–90	
	nr	1	1	0	0	0–98	
Total		464	233	231			

^1^ Includes 39 samples from published reports [11,25].

**Table 3:** MAT results for *L. interrogans* serovar Hardjo from platypuses that were sampled more than once. There were 27 platypuses from the upper Shoalhaven River, NSW, one from the Wollondilly River, NSW and one captive platypus from VIC. Animals are grouped according to whether they had a persistent titre, a titre that resolved, no titre detectable on any occasion, or a result that changed from negative to positive (i.e., that sero-converted).

No. of Times Sampled	No. of Individual Platypuses	Interval between 1st and Last Sampling (Months)		Max. Titre	Change in Titre over Time (No. Doubling Dilutions)	
		Min.	Max.		Min.	Max.
Persistent titre (always >50)						
2	13 ^1^	0.5	59.4	3200	0	2
3	2	15.0	22.3	400	2	2
4	1	12.4	12.4	3200	0	0
5	3	13.1	46.0	3200	1	2
6	2	58.4	70.2	3200	2	5
Titre resolved (>50 then <50)						
2	1	11.7	11.7	3200	7	7
3	1	24.2	24.2	3200	7	7
No titre (always <50)						
2	3	0.9	2.6	<50	-	-
3	2	10.9	11.8	<50	-	-
Seroconverted (<50 then >50)						
2	1 ^2^	17	17	1600	5	5

^1^ Includes one platypus from the Wollondilly River that was sampled two weeks apart [25]. ^2^ Captive platypus.

**Table 4:** Association between lactation status and *L. interrogans* serovar Hardjo MAT results in adult female platypuses captured in the Shoalhaven River during the breeding season (November to February) between 1985 and 1994. Lactation status was determined whenever blood samples were collected.

Lactation	No. Animals Tested ^1^	MAT			Fisher’s Exact Test
		No. NEG	No. POS	Percent Positive	
No	17	2	15	88.2	*p* = 0.52
Yes	10	0	10	100	
	27	2	25		

^1^ 24 individuals, three of which were assessed in more than one breeding season.

**Table 5:** Tests conducted for leptospirosis in four platypuses that drowned in fishing nets in NSW. FA, female adult; MA, male adult; MJ, male juvenile. Neg, negative; Pos, positive; nt, not tested.

Platypus Number	Location (River)	Date Necropsied	Sex and Age	MAT Titre *L. interrogans* Serovar Hardjo ^1^	Histopathology		Dark-Field Examination of Urine for Leptospires	Leptospiral Culture of Kidney/Urine/Blood
					Interstitial Nephritis	Warthin Starry Stain for Leptospires		
RN85/756	Abercrombie	Apr 1985	MJ	<100	Neg ^2^	Neg	nt	Neg/Neg/Neg
RN86/112-1	Murrumbidgee	Jan 1986	MA	100	Pos ^2^	Pos	nt	Neg/nt/nt
RN86/112-2	Murrumbidgee	Jan 1986	MA	300	Pos ^2^	Pos	Neg	Neg/Neg/nt
RN86/1112	Queanbeyan	May 1986	FA	100	Pos	Pos	nt	Neg/nt/nt

^1^ Result obtained in 1985. ^2^ There were also viral inclusion bodies in the renal tubular epithelium [45].

**Table 6:** Blood samples from platypuses in the upper Shoalhaven River that were subjected to leptospiral culture at the time of collection. The lab accession numbers match the data in Table S1.

Lab Accession Number	Date Collected	No. Animals Tested
RN85/518	Mar 1985	41
RN85/2803	Dec 1985	13
RN86/595	Mar 1986	32
Total		86

**Table 7:** Serological results from cattle in ten herds located on eight farms. Cattle grazed on pastures and had direct access to the upper Shoalhaven River or Jerrabattgulla Creek, NSW. Each row represents a separate herd.

Farm (Herd)	Location	Date Collected	No. Samples	No. MAT Positive for *L. interrogans* Serovar Hardjo	Seroprevalence %	95% c.l. %	Highest Titre
1a(1)	Shoalhaven R	Jun-85	50	24	48	34–63	300
1b (2)	Shoalhaven R	Jun-85	10	0	0	0–31	<100
2 (3)	Shoalhaven R	May-85	43	1	2.3	0–12	100
2 (4)	Shoalhaven R	May-85	40	0	0	0–9	<100
2 (5)	Shoalhaven R	May-85	80	1	1.3	0–7	100
3 (6)	Jerrabattgulla Ck	Jul-85	46	4	8.7	2–21	100
4 (7)	Jerrabattgulla Ck	Nov-85	41	0	0	0–9	<100
5 (8)	Jerrabattgulla Ck	Aug-85	79	7	8.9	4–17	2700
6 (9)	Jerrabattgulla Ck	Jun-85	64	11	17	9–29	300
7 (10)	Shoalhaven R	Jun-85	62	0	0	0–6	<100

## Data Availability

Data for serum samples from platypuses are provided in Supplementary Table S1. Data for serum samples from cattle are provided in Supplementary Table S2.

## Supplementary Materials

The following supporting information can be downloaded at: https://www.mdpi.com/article/10.3390/ani14192834/s1, Table S1: platypus data 17-7-24; Table S2: cattle data 17-7-24.

## Appendix A

**Table A1:** Recorded movements of individual platypuses in rivers in NSW and VIC based on formal research. AT, acoustic tracking; M-R, mark–recapture; RT, radiotracking.

River Basin	River/Creek	Jurisdiction	Movements (kms)			Methodology	Reference
			Range	Mean	Max		
Border Rivers	Severn River	NSW	0.4–5.9	-	11.1 *	AT	[119]
Shoalhaven River	Shoalhaven River	NSW	0.2–5.6	1.3	5.6	M-R, RT	[79,120]
Snowy River	Thredbo River	NSW	0.4–2.3	-	2.3	RT	[79,80,81,82,83,84,85,86,87,88,89,90,91,92,93,94,95,96,97,98,99,100,101,102,103,104,105,106,107,108,109,110,111,112,113,114,115,116,117,118,119,120,121]
Snowy River	Snowy River	NSW	0.2–7.1	-	7.08	AT	[122]
Goulburn River	Goulburn River	VIC	0.4–2.6	-	-	RT	[123]
Upper Murray River	Mitta Mitta River	VIC	1.8–8.5	-	8.5	AT	[124]
Yarra River	Badger Creek	VIC	0.3–2.3	1.4	2.3	M-R, RT	[53]
Yarra River	Badger Creek	VIC	2.7–7.0	2.0	15.0 *	M-R, RT	[125]
Yarra River	Yarra River, other	VIC	2.9–7.3	4.6	10.4 *	RT	[126]

* outliers.
